# A Systematic Review on the Advancements in Remote Sensing and Proximity Tools for Grapevine Disease Detection

**DOI:** 10.3390/s24248172

**Published:** 2024-12-21

**Authors:** Fernando Portela, Joaquim J. Sousa, Cláudio Araújo-Paredes, Emanuel Peres, Raul Morais, Luís Pádua

**Affiliations:** 1 Centre for the Research and Technology of Agro-Environmental and Biological Sciences, University of Trás-os-Montes e Alto Douro, 5000-801 Vila Real, Portugal; fernandoportela@utad.pt (F.P.); eperes@utad.pt (E.P.); rmorais@utad.pt (R.M.); 2 proMetheus—Research Unit in Materials, Energy and Environment for Sustainability, Escola Superior Agrária, Instituto Politécnico de Viana do Castelo, 4900-347 Viana do Castelo, Portugal; cparedes@esa.ipvc.pt; 3 Agronomy Department, School of Agrarian and Veterinary Sciences, University of Trás-os-Montes e Alto Douro, 5000-801 Vila Real, Portugal; 4 School of Science and Technology, University of Trás-os-Montes e Alto Douro, 5000-801 Vila Real, Portugal; jjsousa@utad.pt; 5 Centre for Robotics in Industry and Intelligent Systems (CRIIS), INESC Technology and Science (INESC-TEC), 4200-465 Porto, Portugal; 6 CISAS—Center for Research and Development in Agrifood Systems and Sustainability, Instituto Politécnico de Viana do Castelo, 4900-347 Viana do Castelo, Portugal; 7 Institute for Innovation, Capacity Building and Sustainability of Agri-Food Production, University of Trás-os-Montes e Alto Douro, 5000-801 Vila Real, Portugal

**Keywords:** precision viticulture, proximal sensing, multispectral, hyperspectral, thermal infrared, powdery and downy mildew, esca complex, viral diseases, *Flavescence dorée*, artificial intelligence

## Abstract

Grapevines (*Vitis vinifera* L.) are one of the most economically relevant crops worldwide, yet they are highly vulnerable to various diseases, causing substantial economic losses for winegrowers. This systematic review evaluates the application of remote sensing and proximal tools for vineyard disease detection, addressing current capabilities, gaps, and future directions in sensor-based field monitoring of grapevine diseases. The review covers 104 studies published between 2008 and October 2024, identified through searches in Scopus and Web of Science, conducted on 25 January 2024, and updated on 10 October 2024. The included studies focused exclusively on the sensor-based detection of grapevine diseases, while excluded studies were not related to grapevine diseases, did not use remote or proximal sensing, or were not conducted in field conditions. The most studied diseases include downy mildew, powdery mildew, *Flavescence dorée*, esca complex, rots, and viral diseases. The main sensors identified for disease detection are RGB, multispectral, hyperspectral sensors, and field spectroscopy. A trend identified in recent published research is the integration of artificial intelligence techniques, such as machine learning and deep learning, to improve disease detection accuracy. The results demonstrate progress in sensor-based disease monitoring, with most studies concentrating on specific diseases, sensor platforms, or methodological improvements. Future research should focus on standardizing methodologies, integrating multi-sensor data, and validating approaches across diverse vineyard contexts to improve commercial applicability and sustainability, addressing both economic and environmental challenges.

## 1. Introduction

Viticulture holds a significant position in global agricultural activities for its economic and cultural importance. In 2023, approximately 7.2 million hectares of grapevines (*Vitis vinifera* L.) were cultivated worldwide, producing around 237 thousand hectolitres of wine, according to the International Organisation of Vine and Wine (OIV) [1]. Despite its importance, this sector faces numerous challenges, including climate variability [2], the presence of pests and diseases [3,4], and broader socio-economic factors influencing wine production and marketing [5].

The wine industry is highly sensitive to climate change, which directly affects grape quality, production levels, and vineyard viability [6]. Global increases in average temperature, extreme weather events, and changes in precipitation patterns exacerbate these challenges across all wine-growing regions [7]. A noticeable consequence of these changes is the longer duration of warm periods [8], which can degrade grape quality, including flavour and texture [9]. Prolonged heat exposure can cause grape dehydration and loss of acids and sugars, ultimately reducing wine quality [10]. Rising temperatures also disrupt the grapevine growth cycle, leading to premature bud break and harvest [9], which may result in early ripening and lower concentrations of sugars and acids at harvest [11,12]. Additionally, changes in precipitation patterns also influence grapevine health. Extended drought periods can cause grapevine dehydration and water stress [13], increasing vulnerability to diseases and pests [14]. On the other hand, intense and frequent precipitation can lead to grape rot, adversely affecting both quality and yield of the harvest [15].

Among these challenges, grapevine diseases are particularly damaging, reducing productivity and grape quality with direct implications for wine production [16]. These diseases, caused by fungi [17], bacteria [18], viruses, and nematodes [15], affect every part of the plant, including leaves, fruits, trunks, branches, and roots [19]. Effective disease management is, therefore, crucial for maintaining grapevine health and ensuring high-quality grape production.

Traditional approaches to grapevine disease detection rely on field observations and laboratory analysis [20]. Field observations require regular inspection of leaves, bunches, and other plant parts for visible symptoms [21], such as spots, discoloration, and deformities [15]. These visual inspections typically compare diseased plants with healthy ones or known symptoms and analyze characteristics such as plant height, foliage color and shape, branch density, and root system changes. While visual observations help to determine the cause—whether nematode, fungus, bacterium, or virus [22]—they can be subjective and prone to misinterpretation due to overlaps with nutrient deficiencies, natural phenological changes, or damage from climatic events or machinery [22].

Molecular laboratory techniques, such as ELISA (enzyme-linked immunosorbent assay) [23] and PCR (polymerase chain reaction) [24], have become standard tools for detecting plant pathogens. ELISA identifies disease-associated antigens using specific antibodies, while PCR amplifies DNA sequences of the disease-causing microorganism [25]. Although highly reliable, these methods are often time- and labour-intensive, requiring pathogen-specific reagents, which limits their feasibility for large-scale or real-time applications. While traditional approaches to grapevine disease detection rely on labour-intensive field observations and molecular techniques, their limitations in scalability and real-time application highlight the need for innovative sensor-based technologies. Emerging advancements in sensing and AI-based systems may provide solutions to address these challenges, enabling early and accurate disease detection. Additionally, climate-based monitoring approaches, such as the “rule of three 10” (temperatures above 10 °C, 10 mm of precipitation, and grapevine shoots at least 0.10 m long), are also used to predict conditions favourable to diseases like downy and powdery mildew [26]. However, each of these methods has specific advantages and limitations, and are often used in combination to detect and prevent vineyard diseases [27].

The growing demand for faster, more efficient, and non-destructive methods has driven innovation in vineyard disease detection. Early efforts focused on laboratory-based applications, where sensors were used to analyze infected plant material collected from the field to confirm disease presence. Among the initial applications, spectroradiometry was used to identify spectral differences between healthy and diseased leaves across wavelengths from 350 nm to 2400 nm [28,29]. These approaches demonstrated that specific wavelength ranges, particularly between 400 and 1000 nm, were effective in distinguishing disease symptoms [30]. However, the focus of vineyard disease detection has shifted towards the use of digital technologies, including artificial intelligence (AI) [31], Machine Learning (ML) [32], digital image processing, and statistical analysis [33,34]. These methods improve the capabilities of sensor-based systems, enabling faster, more accurate, and efficient disease detection, which supports more effective vineyard management.

Recent advancements in vineyard disease detection focus on scalable and accessible methods, integrating the use of remote [35,36] and proximal sensing [37] technologies. Sensors for vineyard disease detection are becoming prominent in viticulture research, offering early disease detection capabilities that enable precise and proactive vineyard management. Several types of sensors are used, including optical sensors [38,39,40], such as thermal infrared (TIR) [41], multispectral (MSP) [42,43], hyperspectral (HYP) [33,44,45], and RGB [46]. These sensors use distinct properties of electromagnetic radiation to capture disease symptoms, facilitating effective disease identification and monitoring under diverse vineyard conditions.

This review aims to evaluate recent advancements in grapevine disease detection technologies, with a focus on remote and proximal sensing methods, to identify existing research gaps and future opportunities for optimizing vineyard management. By conducting a systematic literature review, based on queries in two different databases, this article identifies and analyses relevant publications focused on grapevine disease detection, using remote and proximal sensing. A bibliometric analysis was performed to examine the main topics, including the diseases addressed, affected plant parts, sensor and platform types, and data treatment methodologies. Furthermore, current limitations and future perspectives for advancing grapevine disease detection technologies are discussed.

## 2. Systematic Review Methodology

### 2.1. Review Methodology

This systematic review article was conducted following the Preferred Reporting Items for Systematic Reviews and Meta-Analyses (PRISMA) protocol [47,48], which ensures transparency and rigor in the identification and selection of relevant studies (PRISMA 2020 abstract checklist in Table S1 and PRISMA 2020 checklist in Table S2). The methodology followed a structured process to identify publications focused on grapevine disease detection using remote and proximal sensing techniques. Two major bibliographic databases, Scopus and Web of Science (WoS), were queried to capture a broad range of relevant publications. To minimize bias, searches on both platforms were performed independently, ensuring a uniform process at all review stages, reducing individual biases, and broadening the scope of the evidence identified.

The search queries were designed to include terms found in the title, abstract, and keywords of the manuscripts. These terms spanned three main categories: (1) the crop under study (grape, grapevine, *Vitis vinifera*, vineyard, and viticulture); (2) grapevine diseases (disease, mildew, esca, trunk disease, rot, botrytis, *Flavescence*, virus, leafroll disease, fungi); and (3) the technologies analyzed (remote sensing, proximal sensing, UAV, unmanned aerial vehicle, UAS, unmanned aerial system, drone, satellite, aircraft, spectrometer, multispectral, vegetation index/indices, hyperspectral, thermal infrared, thermal, ground sensing, optical device, optical sensing, thermography, RGB). Combining these categories ensured the capture of a dataset of relevant studies; the queries performed for each database are available in Table S3.

**Figure 1:**
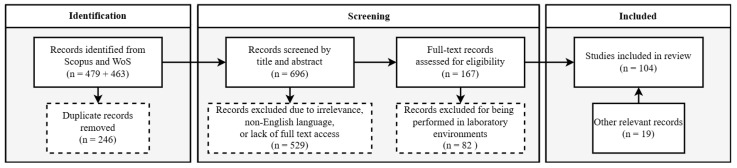
PRISMA flowchart illustrating the systematic review process for identifying and selecting studies on grapevine disease detection and/or monitoring using sensor-based technologies under field conditions.

The search query, conducted on 25 January 2024 and updated on 10 October 2024, resulted in a combined total of 942 manuscripts: 479 from Scopus and 463 from WoS. After removing 246 duplicates, 696 unique manuscripts were selected for the screening process. During the screening process, titles and abstracts fitting the topic of grapevine disease using proximity and remote sensing techniques were analyzed, resulting in 167 publications. An eligibility process based on a full-text analysis was then implemented to filter publications not related to field-based disease detection and monitoring, leading to the exclusion of 82 studies that were exclusively laboratory-based. An additional search in Google Scholar resulted in 19 relevant studies, leading to a final dataset of 104 publications for review. Figure 1 provides an overview of the systematic selection process.

The reviewed publications were categorized and analyzed based on three types of scientific publications: journal articles, conference papers, and book chapters. Following the final selection, several parameters were extracted, including the approach to disease detection, which was categorized as remote, proximity-based, or a combination of both. The studies were further analyzed to identify the types of sensors used, as well as the type of diseases (fungal, bacterial, and viral) and the specific diseases addressed in each case.

### 2.2. Bibliometric Analysis

**Figure 2:**
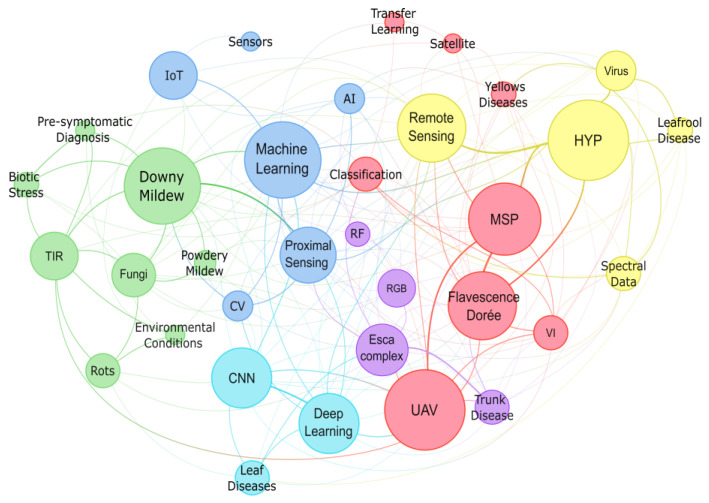
Color-coded keyword clusters from the reviewed studies and their relationships. Created using VOSviewer (version 1.6.20). Each color represents a different cluster. MSP: multispectral; HYP: hyperspectral; VI: vegetation indices; TIR: thermal infrared; IoT: Internet of Things; AI: artificial intelligence; RF: random forest; CNN: convolutional neural network; CV: computer vision.

An analysis of the keywords defined by the authors of the selected studies highlighted several prominent terms, including unmanned aerial vehicles (UAV), ML, downy mildew, deep learning, and convolutional neural networks (CNNs). Additionally, remote sensing and proximal sensing were also frequently mentioned. The most commonly discussed diseases included downy mildew, *Flavescence dorée*, esca complex, yellow diseases, powdery mildew and rot. Among the sensors cited, RGB, MSP, spectroscopy instruments, and TIR sensors were the most frequently referenced. Data analysis techniques, including ML, deep learning, and specific methods such as CNNs and random forests (RF), also stood out, suggesting a growing trend in recent years towards using AI techniques for disease prediction and identification in vineyards. This underscores a change in vineyard research toward adopting advanced technologies for disease monitoring, detection, and prevention. UAVs are particularly essential in remote sensing, while MSP, HYP, and TIR sensors provide detailed and accurate data collection. This data is processed using advanced data analysis techniques, including ML and deep learning, to predict and identify diseases and their symptoms (Figure 2). This approach—combining UAVs, advanced sensors, and AI—offers an efficient and accurate method of managing vineyard diseases, enabling winegrowers to take proactive measures to protect their vineyards.

An analysis of the color-coded clusters in Figure 2 reveals six distinct keyword clusters, each representing unique research themes and methodologies. These clusters provide insights into the diverse approaches and technologies used for grapevine disease detection. The green cluster includes terms such as downy mildew, TIR, fungi, powdery mildew, rots, biotic stress, pre-symptomatic diagnosis, and environmental conditions. It suggests that TIR imaging is mainly applied in detecting fungal diseases like downy mildew and powdery mildew, with pre-symptomatic diagnosis and environmental conditions considered critical for early disease detection and its mitigation.

The blue cluster focuses on ML, proximal sensing, computer vision, AI, sensors, and Internet of Things (IoT), highlighting the role of ML algorithms combined with proximal sensing and advanced sensors for improved disease detection. Computer vision and artificial intelligence techniques are used for data processing and analysis of sensor data, while IoT enables support for real-time monitoring and data integration. The cyan cluster, with fewer keywords including CNN, deep learning, and leaf diseases, highlights the importance of CNNs and other deep learning techniques in identifying and classifying grapevine leaf diseases.

The purple cluster includes terms such as RF, RGB, esca complex, and trunk disease underscoring the use of RF and RGB imagery in detecting symptoms of diseases like esca and other trunk diseases. The red cluster includes MSP, UAV, *Flavescence dorée*, vegetation indices, classification, satellite, transfer learning, and yellow diseases, suggesting that UAVs combined with MSP sensors are used to calculate vegetation indices for monitoring diseases like *Flavescence dorée* and yellow diseases. Additionally, transfer learning and satellite imagery contribute to improved classification accuracy and scalability in remote sensing applications. The yellow cluster includes HYP, virus, leafroll disease, spectral data, and remote sensing, indicating that HYP imaging enables spectral analysis for detecting viral infections and leafroll diseases.

By examining these clusters, it becomes evident that integration of different sensor technologies, imaging methods, and ML models improves the detection and management of grapevine diseases. Additionally, the reviewed studies highlight the role of data processing techniques. This analysis demonstrates how the combination of sensors, platforms, and data processing techniques drives innovation in grapevine disease management. Section 3 explores these platforms, sensors, data processing methods, and grapevine diseases in more detail.

## 3. Overview of Platforms, Sensors, Diseases and Data Processing Techniques

### 3.1. Platforms Capabilities and Applications

Precision viticulture relies on different platforms that facilitate vineyard management by providing diverse types of data with varying resolutions and coverage areas. These platforms include satellites, manned aircraft, UAVs, IoT devices, weather stations, and agricultural machinery, each with its own advantages and limitations.

Satellite remote sensing platforms, despite their lower resolution, are valuable for monitoring large-scale vineyard areas. Typically, their spatial resolution exceeds 10 m, making them suitable to provide a broad overview of vineyard conditions [49], and identify areas of water stress [50] and map variability of plant health and vigour [51]. Although satellite data lacks the granularity required for interventions at the individual plant level, it supports decision-making in irrigation planning [52], is especially useful for large vineyards where timely manual observations are impractical [49], and, by leveraging regular revisit periods, allows temporal analyses to identify changes over time, such as the effects of seasonal climate variations, plant growth, and the development of large-scale problems [53].

Manned aircraft offer the advantage of carrying heavy payloads capable of capturing higher-resolution data than freely available satellite imagery [54]. While satellite-based imaging is constrained by the distance between the platform and the Earth’s surface, resulting in resolutions that can vary from meters to tens of meters per pixel, sensors on manned aircraft can operate closer to the surface, capturing data at sub-meter resolutions [55]. However, this approach is costly and subject to logistical limitations and availability for data acquisition. The higher spatial resolution enables distinguishing grapevines from the surrounding vegetation, a task unfeasible with lower-resolution satellite data [56], allowing identification of subtle variations in plant color and temperature [57], which may indicate issues such as disease presence [58]. Additionally, such data can be useful for assessing water and heat stress across medium-to-large vineyards [59], enabling resource allocation and mitigating environmental impacts [60].

UAVs have become an effective tool in precision viticulture over the past decade [61]. These platforms, which may be designed as fixed-wing or rotary-wing, can be equipped with high-resolution cameras and sensors, such as RGB, TIR, and MSP [62]. UAVs enable the detailed monitoring of crop conditions, facilitating the precise identification of issues such as pests, diseases [63], and water stress [64]. Data from this platform allows making decisions in near real-time, leading to accurate resource management and improved grape quality [65]. Furthermore, UAVs can improve operational efficiency [66], such as the administration of phytosanitary treatments [67,68], by generating prescription maps. This integration of precision viticulture and sustainability supports quality grape production while reducing ecological footprints [69].

Proximity platforms, including agricultural machinery and IoT devices, can be employed standalone or to complement remote sensing methods by providing close-range, high-resolution data. Weather stations and IoT devices can be equipped with sensors designed to measure different environmental parameters. These platforms are versatile and capable of hosting multiple sensors that collect real-time data, for optimizing viticulture practices, improving yields, and ensuring grapevine health [70]. Such platforms also offer integration and connectivity capabilities, enabling better decision-making in vineyard management.

Agricultural machinery such as tractors equipped with sensors serve as dual-purpose platforms, collecting real-time data on plant health, soil properties, and disease symptoms [71] while performing vineyard operations [72]. In addition, smartphones have become accessible and versatile tools for close-range data collection, allowing users to capture images of disease symptoms in crops. Equipped with high-quality cameras and applications, smartphones can record visual symptoms on leaves, stems, or fruits, supporting early disease detection and documentation [73]. Advanced smartphone applications can use ML algorithms to analyze these images in real-time, often providing diagnostics directly to the user. The combination of agricultural machinery and smartphones offers benefits for managing crop health. While agricultural machinery covers productivity areas, smartphones facilitate flexible and user-driven data acquisition, achieving a balance between wide-scale monitoring and detailed analysis [72].

The platforms described in this section demonstrate the diversity of tools available for precision viticulture. Table 1 provides a summary of both the remote and proximal platforms and equipment, detailing their coverage, availability, costs and capacity.

### 3.2. Sensors for Grapevine Disease Detection

A variety of sensors can be used for grapevine disease detection, offering data across the electromagnetic spectrum and other vineyard parameters. These sensors include devices such as RGB (Red, Green, Blue), TIR, as well as MSP, spectroscopy instruments including HYP imaging systems and handheld spectroradiometers, alongside sensors embedded in IoT devices and weather stations. These sensors can collect vineyard data related to soil, plants, and climate conditions, interconnected through the Internet [74,75].

RGB sensors operate within the visible spectrum, capturing variations in light intensities across the three primary color bands [75]. In viticulture, RGB sensors can be used to monitor leaf color, vigour, and canopy density. These sensors enable the identification of visual symptoms of stress, discoloration, and variations in foliage density, which are early indicators of plant health issues. By analyzing these images, vegetation indices can be assessed, assisting in irrigation, fertilisation, and pest control, promoting more efficient vineyard management [76].

TIR sensors measure the radiation emitted by plants and soil, converting it into temperature data. In viticulture, TIR sensors are used for assessing grapevine surface temperature, which directly relates to plant water status. For example, increased leaf temperature indicates reduced transpiration caused by water stress [64]. This data supports efficient irrigation strategies, ensuring optimal water use [77].

MSP sensors capture data across multiple regions of the electromagnetic spectrum, typically covering the visible and near-infrared bands. These sensors detect subtle variations in plant reflectance, enabling assessments of vegetative vigour, chlorophyll content, and overall plant health [74]. They can also assist in the early detection of pests and diseases by identifying changes indicative of stress. This data supports the creation of vineyard variability maps, which guide localised input applications, improving sustainability and production efficiency [78].

Spectroscopy instruments capture reflected light from plants and soil across a wide range of wavelengths, with a high spectral resolution. Analysing this data provides detailed information on chemical and biochemical composition of grapevines, such as nutrient levels, pigments, and stress indicators [79]. HYP imaging systems can cover hundreds of spectral bands, enabling the identification of specific compounds and aiding in the assessment of grape ripeness. This precision allows optimizing vineyard management, determining ideal harvest timing, and improving input efficiency [80].

IoT devices and weather stations equipped with sensors monitor critical environmental and soil parameters, including air temperature, humidity, wind speed, precipitation, soil moisture and temperature, and solar radiation [81]. These sensors provide real-time data for analyzing vineyard microclimates and grapevine growth conditions. By integrating this data with vineyard management systems, winegrowers can predict the occurrence of disease outbreaks and adapt to climate changes, ultimately improving production efficiency and resilience to climatic changes [82]. Table 2 presents the viticulture applications of each sensor and the types of measurements they provide.

### 3.3. Characteristics and Impacts of Grapevine Diseases

**Figure 3:**
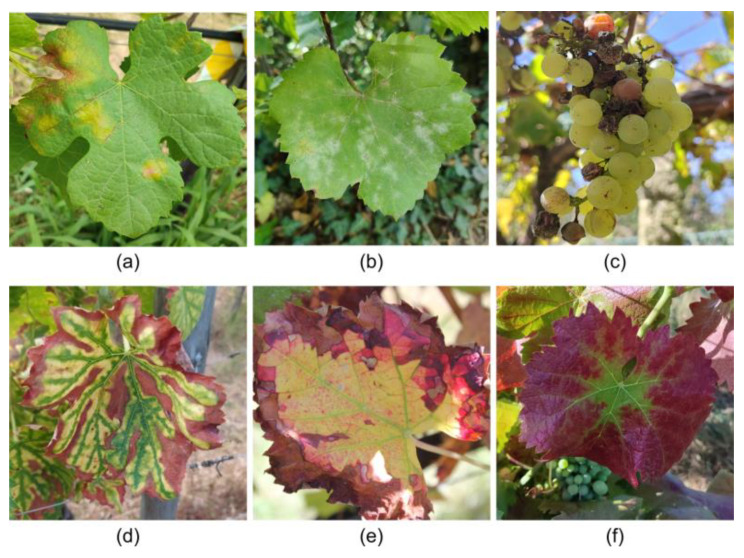
Visual symptoms of grapevine diseases: (**a**) downy mildew; (**b**) powdery mildew; (**c**) bunch rots; (**d**) esca complex; (**e**) *Flavescence dorée*; and (**f**) viral diseases.

Grapevines are affected by a wide variety of diseases that impact productivity and grape quality. These diseases can be categorized into fungal, bacterial, and viral types. Figure 3 illustrates some of the primary symptoms of the diseases addressed in this subsection.

Among the fungal diseases, downy mildew (Figure 3a), caused by *Plasmopara viticola*, is one of the most detrimental grapevine diseases, thriving in humid environments with mild temperatures (15–25 °C), with leaf wetness being essential for spore germination [83]. Symptoms manifest as oily spots on the upper leaf surface that evolve into necrotic lesions, and in severe cases, a white sporulating layer forms on the underside of the leaves. The disease can cause yield losses by inducing fruit shriveling and premature drop [84].

Powdery mildew (Figure 3b), caused by *Erysiphe necator*, prospers in warm, dry climates (20–30 °C) [85] and, unlike downy mildew, does not require leaf wetness for its proliferation. It manifests as a white-grey powdery coating on the leaves, stems, and berries, which inhibits photosynthesis and transpiration, reducing vegetative growth and grape quality. Affected grapes may crack and develop an unappealing appearance, impacting both grape taste and wine production [86]. The main control techniques of downy mildew and powdery mildew are the use of weather stations and predictions [37] to identify optimal infection conditions and the subsequent application of contact or systemic fungicides, which can be chemical or biological [87].

Rots, such as grey rot caused by *Botrytis cinereal* (Figure 3c), are a significant concern in regions with high humidity and precipitation. Grey rot primarily affects grapes, causing decomposition that compromises quality, reducing harvest yield, with excessive proliferation negatively affecting the aroma and wine flavor [88]. Black rot (*Guignardia bidwellii*), which is particularly harmful in warm and humid climates. This fungus survives on infected plant debris, acting as an inoculum for future infections. On the berries, the infection leads to black spots, causing the fruit to shrivel and mummify, resulting in yield reduction. Control measures include removing infected plant debris, pruning to improve air circulation, and applying preventative fungicides [89]. Sour rot, caused by an interaction between yeasts, bacteria, and fungi, is common under high humidity conditions during ripening, causing severe losses due to vinegary odor and berry splitting. White rot (*Coniella diplodiella*) leads to watery rot in grapes that can destroy entire clusters if not controlled. These diseases require integrated management, involving the removal of infected plant material, control of insect vectors, and the use of preventative fungicides to maintain production quality and sustainability [90].

Esca complex (Figure 3d), caused by a combination of fungi including *Phaeoacremonium aleophilum* and *Phaeomoniella chlamydospore*, is a set of trunk diseases that affect mature grapevines, leading to a progressive decline and, ultimately, the plant’s death. Pruning wounds often serve as entry points for these pathogens [91]. Symptoms vary and include chlorotic streaks on the leaves, vascular discoloration, necrosis, and, in advanced stages, “apoplexy”, where the plant experiences sudden death. The disease can remain latent for years before symptoms become visible, making early diagnosis difficult [19].

Among bacterial diseases, *Flavescence dorée* (Figure 3e) is particularly severe. This disease is caused by phytoplasmas that disrupt the vascular system [92], leading to yellowing and curling of the leaves, stunted shoot growth, and necrosis of inflorescences. These symptoms result in yield reductions and often plant death. The disease spreads rapidly through insect vectors, primarily the leafhopper *Scaphoideus titanus*. Effective management relies on rigorous vector control measures, which are crucial to prevent widespread outbreaks [93].

Viral diseases, such as Grapevine Leafroll-associated Virus (GLRaV) 1, 2 and 3, Grapevine virus A (GVA)*,* and Grapevine vein clearing virus (GVCV), present additional challenges. GLRaV symptoms vary depending on the virus but often include leaf discoloration, rolling, stunted growth, and reduced fruit yield. Leafroll disease, the most common symptoms are red or yellow discoloration accompanied by rolling leaf margins. This reduces photosynthesis, leading to decreased plant vigor (Figure 3f). Fruits may exhibit uneven ripening and reduced sugar content, compromising wine quality [94]. GVA is part of the *Betaflexiviridae* family and the *Vitivirus* genus and is commonly associated with grapevine trunk diseases (GTD) such as rugose wood complex. Symptoms include bark cracking and damage to vascular tissues, reducing grape yield and quality. While some grape varieties may remain asymptomatic, GVA can lead to reduction in both the quantity and quality of fruit produced in susceptible varieties [95]. GVCV causes vein clearing in leaves, which can progress to deformities and decreased plant vigor. GVCV’s impact on commercial vineyards can be severe, reducing both fruit quality and yield. While research on GVCV’s transmission pathway continues, it is believed to spread through grafting or insect vectors [96]. A summary of the diseases addressed in this subsection is presented in Table 3.

### 3.4. Data Processing and Analysis Techniques

The use of data collected by sensors depends on robust processing and analysis techniques. These methods transform raw data into useful information for more precise vineyard management. Pre-processing steps, such as radiometric and geometric corrections, are essential for ensuring data quality. Data collected from sensors coupled with the different remote sensing platforms, particularly UAVs and manned aircraft, undergoes pre-processing to create orthorectified [97] and radiometric raster products. For example, UAV-based MSP and HYP data acquisition should ensure radiometric calibration to ensure the reflectance of the scene radiance [98,99]. Geometric corrections, including the use of real-time kinematic (RTK) position systems [100] or ground control points (GCPs) [101,102], are necessary for accurate representation and multi-temporal data alignment.

Additionally, spectral data can be combined through arithmetic operations to calculate vegetation indices [79]. For three-dimensional vineyard analysis, photogrammetric processing techniques using Structure-from-Motion (SfM) are applied to generate point cloud data. This data is further used to compute orthorectified raster products such as orthophoto mosaics, digital surface models (DSMs), and Digital Terrain Models (DTM). These can be used to generate canopy surface models (CSMs) or canopy height models (CHMs), which are important in evaluating canopy structure and filtering non-grapevine pixels [103].

For IoT and weather stations, data processing is relatively simpler, as they primarily provide point-based environmental and soil measurements. These datasets are often integrated with statistical and ML models to predict vineyard conditions and support decision making [104]. Techniques such as regression analysis are used to understand the relationships between environmental variables and vineyard performance. Moreover, imagery captured at proximal ranges can be used to build datasets for specific applications, such as digital image processing and computer vision techniques and training classifiers using AI techniques. These techniques can be used for segmentation (highlighting meaningful parts of an image), classification (assigning labels to images or specific regions), and object detection (identifying and locating classes of objects within an image).

To evaluate the accuracy of disease detection methods, several statistical measures are used according to the classification result. These include precision, which is the ratio of correctly predicted positive observations (true positives) to the total predicted positives (true positives + false positives). Recall is the ratio of correctly predicted positive observations to all observations in the actual class (true positives + false negatives). The F1-Score is the harmonic mean of Precision and Recall, and is useful when there is a need to balance the two metrics and to measure inter-class variability. Overall accuracy is the ratio of correctly predicted observations to the total observations. The Area Under the ROC Curve (AUC-ROC) is a performance measurement for classification problems at various threshold settings, indicating the model’s capability to distinguish between classes. Intersection over Union (IoU) is used for object detection and segmentation tasks. For segmentation, IoU measures the overlap between the predicted and ground truth while for object detection tasks it measures the overlap between the predicted bounding box and the ground truth bounding box.

Regression models are used to predict the value of a dependent variable based on one or more independent variables. The most commonly used regression model is linear regression, which assumes a linear relationship between variables [105]. Logistic regression, another technique, is applied when the dependent variable is categorical. Both approaches aim to minimize the difference between predicted and observed values, resulting in an effective predictive model [106]. Principal Component Analysis (PCA) is a dimensionality reduction technique that simplifies datasets with many correlated variables by transforming them into principal components, which capture the maximum variance in the data using the fewest components [107]. Linear Discriminant Analysis (LDA) can be used for classification and dimensionality reduction; it maximizes the separation between different classes by identifying directions that increase the distance between class centers while minimizing intra-class variance. LDA is useful when the classes follow Gaussian distributions and are linearly separable [108]. Standard Normal Variate (SNV) is a pre-processing technique that can be applied to spectroscopy data to remove effects of light scattering or systematic noise. SNV application normalizes the data by subtracting the mean and dividing by the standard deviation, improving the quality of subsequent models [109]. Partial least squares discriminant analysis (PLS-DA) is an extension of the Partial Least Squares (PLS) technique and can be used for data classification. While PLS models relationships between independent and dependent variables, PLS-DA also discriminates between different classes, making it effective for analyzing multivariate data [110].

The application of big data analytics and AI techniques can be employed in viticulture, providing approaches for monitoring soil conditions, monitoring plant health, and predicting pests and diseases. AI-based methods are able to process large volumes of data from various sources, such as environmental sensors and satellite or UAV imagery, to deliver precise real-time information [111]. This contributes to better decision support regarding critical aspects such as irrigation and pesticide use, optimizing production and ensuring grape quality. ML techniques are effective at identifying complex patterns in agricultural data. For example, ML algorithms can recognize subtle correlations between climatic conditions, soil types, and management practices, helping to forecast grape yield and quality. One application of ML in viticulture is predicting vineyard water stress based on climate and soil moisture data, which supports more efficient irrigation [112]. RF, a supervised learning method, has proven useful in analyzing viticultural data, such as predicting diseases like downy mildew based on variables like temperature and humidity. The resulting models support decision-making, promoting sustainable vineyard management and improving plant health [113,114]. Artificial Neural Networks (ANNs) enable the analysis of large, complex datasets, facilitating the prediction of climate changes and their effects on grapevines. ANNs can detect hidden patterns in environmental data, optimizing processes such as irrigation and pest control.

Deep Neural Networks (DNNs) and CNNs are effective at detecting diseases and pests using imagery data. For instance, CNNs like VGG-19 [115] and Inception-v3 [116] have shown success in classifying disease symptoms. VGG-19 has more simplicity in its architecture, which provides feature extraction capabilities, though it requires significant computational resources. Inception-v3, on the other hand, uses an inception module that allows the network to learn multi-scale features in parallel, which improves the ability to detect patterns at various scales with greater efficiency. These types of models often benefit from optimization techniques such as transfer learning [117], where pre-trained networks are fine-tuned on specific datasets such as for grapevine disease, improving the performance and reducing training time. Recurrent Neural Networks (RNNs) are used for time series forecasting, enabling proactive vineyard management [118]. Object detection algorithms like YOLO (You Only Look Once) are employed in viticulture to monitor vineyard integrity in a quick and efficient manner, supporting real-time decision-making, which is crucial in dynamic agricultural environments [119].

## 4. Results

### 4.1. Overview of the Reviewed Studies

**Figure 4:**
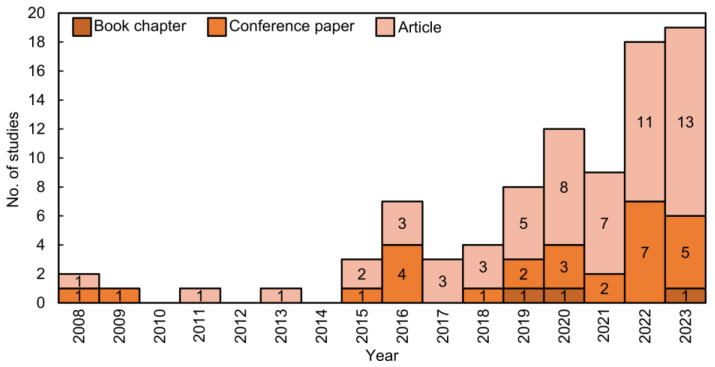
Annual distribution of the identified publications on grapevine disease detection (2008–2023), categorized by manuscript type.

An analysis of the studies published in each year (Figure 4) reveals that from 2015 to 2023, there was an increase in the number of publications related to vineyard disease detection: 25 conference articles and 56 journal articles. Before 2015, only five relevant publications were identified in the bibliography. In 2016, there was a peak with seven publications: three journal articles and four conference papers. In 2015 and 2017, three studies were published in each year. From 2018 to 2023, there was a significant increase in publications, with a slight decrease observed in 2021. In 2019, 2020, and 2023, one book chapter was published each year, with 2023 also marking the highest number of publications (19). As of the query date in 2024, four conference articles and 12 journal articles have already been published, nearly matching the 2023 total, reflecting the sustained and growing interest in this topic.

When analyzing by type of sensors used, RGB, MSP, thermal, HYP, spectroradiometer (both in the category of spectroscopy instruments) and weather stations stand out. Of the reviewed publications, 48 studies (36%) analyzed RGB data, followed by 26 studies (around 19%) using MSP sensors. Data from IoT devices was used in 16 studies (12%), mostly in conjunction with remote sensing and/or proximity sensors. Spectroscopy instruments were used in 25 studies (19%), and TIR sensors in 10 studies (7%). The remaining 7% represent nine types of sensors, such as LiDAR, fluorescence sensors, and RGB-D sensors.

A trend identified in this review was the integration of multiple sensor technologies. Among the reviewed studies, 79 (76%) used at least two types of sensors, with 21 studies integrating two types, three studies using three types, and one study using four types of sensors. This multi-sensor approach highlights the complexity of grapevine disease detection and underscores the value of combining complementary data sources to improve the accuracy and monitoring of grapevine diseases.

### 4.2. Overview of Sensors Used in Proximal and Remote Sensing

**Figure 5:**
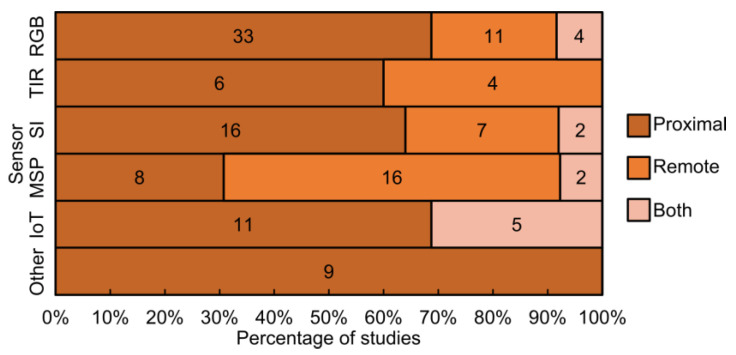
Distribution of sensor use based on proximal or remote sensing studies. TIR: thermal infrared; SI: spectral instruments; MSP: multispectral; RGB: red, green, blue; IoT: Internet of Things.

The majority of the reviewed studies employed proximal sensing techniques for analyzing grapevine diseases, as shown in Figure 5. Specifically, eight studies used data from both proximity sensors and remote sensing platforms (8% of the total). A total of 29 studies (28%) used only remote sensing data, indicating a slight trend towards the use of remote sensors for grapevine disease identification in recent years. Meanwhile, 67 studies (64%) relied only on data from proximal sensing. These results can be related to the challenges of detecting the symptoms of certain diseases using only remote sensing platforms.

Among the 67 studies using proximal sensing (Figure 5), RGB sensors were the most frequently used, featuring in 33 studies (69% of total number of articles about this sensor). TIR sensors were addressed in six studies (60%), while spectroscopy instruments, which include field spectroradiometers, were used in 16 studies (64%). These sensors primarily captured data of specific locations within the grapevine canopy. MSP sensors were used in eight studies (31%), and IoT devices were reported in 11 studies (69%), typically in combination with other sensor types. Other sensors, including fluorescence, LIDAR, and 3D sensors, were used in nine studies.

In remote sensing studies, RGB sensors were used in 11 studies (23%), often in combination with other sensors. TIR sensors were used in four studies (40%). Spectroscopy instruments, namely HYP sensors, were used in seven studies (31%). MSP sensors are used in 16 studies (61%), becoming a primary tool for grapevine disease detection and monitoring using remote sensing platforms.

Additionally, in the studies combining both proximal and remote sensing approaches, four used RGB sensors (8%), two studies used HYP, and two studies used MSP. Spectroscopy instruments are used in five studies, and four studies implemented IoT technology. There is a slight trend towards incorporating data collection using aerial, and spaceborne platforms such as UAVs and satellites into systems designed to assist winegrowers. When analyzing the type of remote sensing platform used, UAVs were the most common, being included in 30 studies. This suggests that UAVs have become the primary remote sensing platform for grapevine disease studies. Satellites were used for disease identification in three studies, while manned aircraft, despite their capacity for collecting high-resolution data over large areas, were used in only four studies.

### 4.3. Techniques and Advancements in Field-Based Grapevine Disease Detection

The grapevine diseases addressed in each study were categorized into four groups based on the primary affected plant parts: trunk diseases, leaf diseases, fruit diseases, and unspecified diseases. Although some trunk and fruit diseases may manifest symptoms on the leaves, they were classified according to the primary affected part, such as trunk or fruit. The Esca disease complex is an illustrative example of this phenomenon as it affects leaves and fruits but is considered a trunk disease.

**Figure 6:**
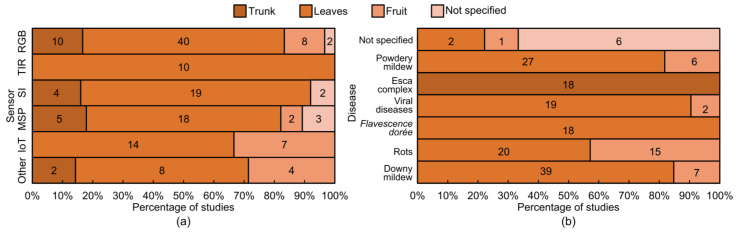
Proportional representation of sensor usage (**a**) and grapevine disease (**b**) based on the type of infection, including trunk diseases, leaf diseases, fruit diseases, and those not specified. TIR: thermal infrared; SI: spectral instruments; MSP: multispectral; RGB: red, green, blue; IoT: Internet of Things.

The reviewed studies show diverse approaches to sensor deployment for disease detection (Figure 6). When analyzing the type of sensor used per disease category, it was found that 40 studies (67%) analyzed leaf diseases using RGB sensors, while 10 studies (17%) addressed trunk diseases. Two studies using RGB sensors did not specify the disease, while eight (13%) addressed fruit diseases. For TIR sensors, 10 studies (100%) focused on leaf diseases. Regarding spectroscopy instruments, four out of the 25 studies addressed trunk diseases, while approximately 76% (19 studies) focused on leaf diseases, and two studies (8%) did not specify the specific disease analyzed. For studies using MSP sensors, five studies (18%) analyzed trunk diseases, two studies (7%) focused on fruit diseases, 18 studies (74%) on leaf diseases, and three were not specified. Among IoT-based studies, 14 studies (67%) used this technology on leaf diseases, and seven studies (35%) on fruit diseases. Additionally, two studies using other sensors addressed trunk diseases, eight focused on leaf diseases, and four on fruit diseases. This distribution is presented in Figure 6a.

Of the publications reviewed, 15% focused on trunk diseases, with 18 addressing the Esca disease complex. Additionally, 13% of the publications examined diseases impacting the grapes, with bunch rot (15 studies) and powdery mildew (six studies) being the primary diseases examined. One study did not specify the disease type but provided details on data analysis and techniques used. Leaf diseases were the most represented, accounting for 67% of total studies. This category included a wide range of grapevine diseases, such as downy mildew (39 studies), powdery mildew (21 studies), *Flavescence dorée* (18 studies), other viral diseases (19 studies), and two unspecified diseases. Lastly, six articles did not specify the disease (Figure 6b). In total, 75 studies focused on a single disease, 13 analyzed two diseases, 11 studies focused on three, three studies addressed four different diseases, and one included five diseases.

#### 4.3.1. Downy Mildew

The early detection of downy mildew (*Plasmopara viticola*) in vineyards is crucial for effective disease management and the reduction of fungicide use. Recent technological advancements have introduced several approaches to address this challenge, including the integration of IoT devices, proximal sensing, TIR imaging, computer vision, and data fusion. The methods make use of environmental monitoring, image analysis, and ML algorithms to improve detection accuracy, optimize interventions, and vineyard management.

IoT-based systems and weather stations are widely used to monitor environmental conditions conducive to downy mildew outbreaks. Hnatiuc et al. [37] implemented a centralized platform integrating sensors to measure air humidity, temperature, leaf wetness, photosynthetically active radiation, sap flow, and soil oxygen, moisture, and temperature. By applying statistical analyses and ML algorithms, their approach successfully predicted infection outbreaks, enabling a reduction in pesticide applications through early interventions. Mezei et al. [120] developed a narrowband IoT (NB-IoT) for hourly monitoring of air temperature, humidity, precipitation, leaf wetness, and wind speed. The developed model issued alerts for primary and secondary infections, showing strong correlations with iMetos^®^ alerts for severe infections and moderate correlations (*R*^2^ = 0.4 to 1) for less severe cases, validating its efficiency for real-time disease forecasting. Similarly, Marcu et al. [121] used sensors to collect atmospheric data and monitor disease presence through a precision agriculture platform. The system gathered air temperature, precipitation, and disease risk indices, providing real-time analysis and visualization of powdery and downy mildew risks. Treatment recommendations based on meteorological data and disease monitoring allowed for effective disease risk management throughout the growing seasons.

Kleb et al. [122] highlighted the importance of canopy-specific data by comparing measurements within the grapevine canopy with those from weather stations. The authors observed discrepancies, such as higher temperatures and reduced leaf wetness within the canopy, which were not reflected in weather station data, highlighting the need to refine forecasting models and fungicide application practices. Sanna et al. [123] further addressed the role of sensor calibration and placement, showing that improper calibration could delay accurate disease forecasts by up to five days.

**Figure 7:**
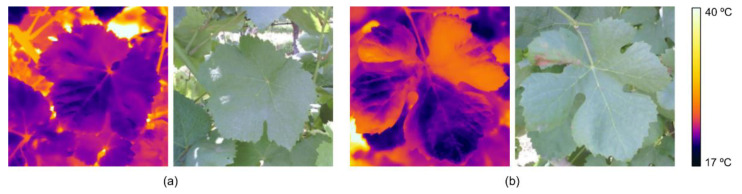
Thermal infrared and RGB images of grapevine leaves showing the thermal behavior of a non-infected leaf (**a**) and a leaf infected with downy mildew (**b**).

Proximal sensing technologies, including thermographic imaging, have demonstrated potential for detecting downy mildew before visible symptoms appear [124]. Cohen et al. [3] employed an active contouring process to analyse thermal images, applying feature selection techniques and models to classify infected and healthy leaves. Among the models tested, SVM achieved an accuracy of 81.6% with an F1 score of 77.5%. Zia-Khan et al. [125] detected a 3.2 °C temperature increase in infected leaves before symptoms appeared, reporting a correlation between disease severity and maximum temperature difference (*R*^2^ = 0.76, *p* ≤ 0.001). Stoll et al. [41,126] used TIR to monitor grapevine leaf responses to water stress and *Plasmopara viticola* infection. The authors observed temperature differences between irrigated and stressed leaves, with reduced stomatal conductance under water stress, enabling early fungal infection detection. These studies suggest that TIR imaging is reliable for early downy mildew detection, while infrared thermography has proven valuable for monitoring plant stress and early pathogen detection, optimizing pesticide application, and improving spraying efficiency, although factors like sun direction and microclimatic conditions require further investigation. Figure 7 provides an example of temperature variations on the leaf surface, comparing a healthy leaf (Figure 7a) to a leaf infected with downy mildew (Figure 7b).

**Figure 8:**
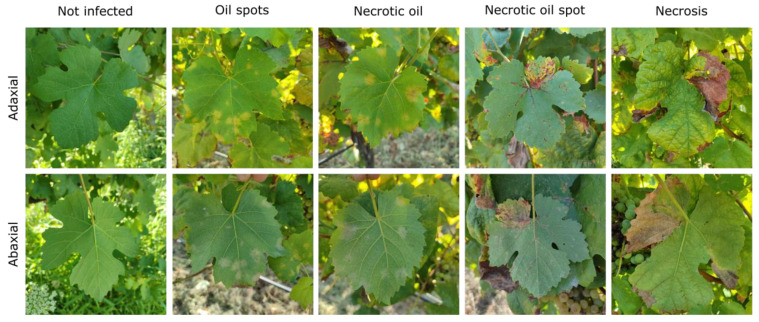
Photographs of different stages of downy mildew in grapevine leaves on both abaxial and adaxial sides of the leaf.

The use of computer vision and deep learning is applied in several studies to assist in downy mildew detection in RGB images and proximal sensing. Liu et al. [40,127] employed a custom all-terrain vehicle (ATV)-based imaging system and proximity RGB cameras with GPS integration; the authors used the Hierarchical Multi-Scale Segmentation with Semantic Attention (HMASS) model. The results showed high correlation with human evaluations (*r* = 0.96) for downy mildew and a mean intersection over union (IoU) of 0.76 for powdery mildew. Poblete-Echeverría et al. [128] achieved strong correlations (*R*^2^ = 0.92, IoU of 0.67) between automated RGB image analysis and expert assessments in field conditions using a SegNet segmentation model. Similarly, Gutiérrez et al. [129] used pre-processing techniques in Hue, Saturation, and Value (HSV) color space to train classifiers, achieving F1-scores of 0.94 and between 0.89 and 0.97 for multi-class and binary classifications, respectively. Abdelghafour et al. [71] used a tractor-mounted RGB industrial camera to capture images for disease classification into seven classes using color-structure representations. The results showed average precision and recall rates of 83% and 76%, respectively. Sandika et al. [46] used an RF classifier with Gray Level Co-occurrence Matrix (GLCM) features to detect diseases such as anthracnose, powdery mildew, and downy mildew, achieving an accuracy of 86% (sensitivity ranging between 71.3% and 87.5% and a specificity ranging between 90.4% and 95%). In turn, Padol and Yadav [130] used K-means clustering to find the diseased region and extracted texture and color features to train an SVM classifier for disease classification (downy and powdery mildew), achieving an accuracy of 88.89% across two disease classes, with 93.33% for downy mildew and 83.33% for powdery mildew. The integration of these techniques in proximal platforms can enable large-scale field monitoring. An example of RGB images that can be used for real-time detection are those shown in Figure 8, where leaves with different stages of the infection are presented.

Other sensors were also used to study this from other perspectives, such as in Latouche et al. [131], who explored the use of violet-blue fluorescence (VBF) as an indicator for detecting and monitoring downy mildew on the adaxial and abaxial surfaces of grapevine leaves, demonstrating that VBF detection occurred earlier than visual assessments in 45% of leaves, compared to only 10% by visual methods. Lefevre et al. [132] classified grape quality using RGB images of crates of grapes and an RF. By applying edge detection and segmentation techniques, the authors developed a quality index that classified grapes in six classes including the presence of diseases (grey rot, powdery mildew, conidia), with implications for automation in the grape production industry.

**Figure 9:**
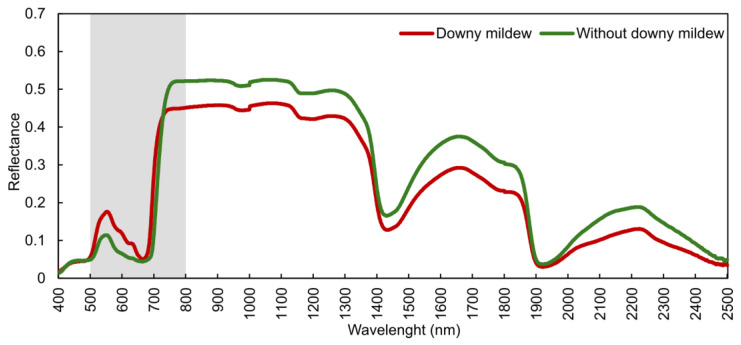
Spectroscopy data of a leaf infected with downy mildew and another without infection. The area highlighted in grey presents differences in the visible and near-infrared parts of the electromagnetic spectrum. Data acquired using ASD FieldSpec 4 (Malvern Panalytical Ltd., Malvern, UK).

Grapevine disease detection through the simultaneous use of multiple proximal data sources can be used for improvements in accuracy and early detection. Yang et al. [133] demonstrated the value of fusing RGB, MSP, and TIR data to train a ShuffleNet V2 to classify foliage images between anthracnose, downy mildew, leafhopper, mites, viral disease, and healthy, achieving an overall accuracy of 96.05%, with RGB imagery showing performance improvements when adding MSP and TIR data. Kanaley et al. [134] used high-resolution satellite imagery (SkySat and PlanetScope, Planet Labs PBC, San Francisco, CA, USA) to identify downy mildew incidence in vineyard row sections. By using RF classifiers trained with vegetation indices and spectral bands, the study highlighted limitations such as cloud cover and spectral resolution, proposing the use of proximal spectrometers for greater precision. The potential of such data is demonstrated in Figure 9, where the spectral signature of downy mildew disease on a grapevine leaf is compared with that of a non-infected leaf. Noticeable differences appear in the visible region (500 to 650 nm), and the red edge and near-infrared parts (700 to 850 nm); wavelengths above 800 nm show higher reflectance values in the leaf without symptoms.

Kerkech et al. [135,136] combined UAV-based RGB and NIR imagery with digital surface models to produce georeferenced disease maps. A SegNet model was trained to classify between shaded areas, soil, healthy grapevines and grapevines, exhibiting symptoms; the segmentation of visible and infrared images achieved an accuracy of 85% and 92%, respectively, in detecting mildew symptoms [135]. In Kerkech et al. [136], the approach was extended by using orthorectified UAV RGB and near-infrared data, along with a DSM to generate a depth map. The authors proposed the VddNet deep learning architecture to produce disease maps from the three UAV-based products, achieving an overall accuracy of 93.72% with an F1-score of 0.93 in the diseased class. Kuznetsov et al. [137] optimized UAV flight paths and used a neural network, achieving a minimum detection accuracy of 91%. The system could monitor up to 2.5 hectares per day. These approaches demonstrate the potential for scalable and efficient vineyard disease management and the potential of multi-sensor UAV-based data fusion for disease mapping.

Integrating data from IoT devices and UAVs can improve/complement vineyard disease detection. Balaceanu et al. [138] proposed a combined integration of UAV imagery with IoT wireless sensor networks (WSN) and ML for disease detection. This approach is applied in Roșcăneanu et al. [139], where IoT platforms and UAVs were used to capture grapevine leaf images and analyze environmental data along with ML algorithms. The authors detected downy mildew, powdery mildew, and grey rot, revealing seasonal patterns in temperature, humidity, and precipitation. Moreover, Ouhami et al. [140] acquired UAV RGB images and meteorological data to predict downy mildew in vineyards, using semantic segmentation techniques to detect grapevine areas in UAV imagery with downy mildew and a long short-term memory (LSTM) recurrent neural network to identify downy mildew in the meteorological data. Kontogiannis et al. [141] proposed a framework for the detection of mildew on grapevines using RGB images captured by IoT devices and UAV imagery. The authors evaluated Faster R-CNN and YOLOv5 models for real-time detection, achieving a mean average precision (mAP) of 0.95 with ResNet-152 for detailed detections, while YOLOv5 obtained a faster processing speed. Patil and Thorat [142] demonstrated the efficacy of Hidden Markov Models (HMMs) for grapevine disease classification (bacterial leaf spot, powdery mildew, downy mildew, anthracnose, bacterial cancer, and rust). The HMM demonstrated high precision (1), recall (0.83), and F-measure (0.91) values, highlighting the robustness in early detection. These methods allow the identification of disease patterns, providing a basis for developing strategies to improve grape quality and optimize production costs, with the complementarity of the integration of low-cost solutions, such as IoT devices [143].

#### 4.3.2. Fruit and Leaf Rots

Fruit and leaf rots, such as black rot (*Guignardia bidwellii*) and *Botrytis cinerea*, are among the most substantial diseases affecting grapevines, often causing economic losses. These fungal infections primarily target grape clusters, leading to complete bunch detonation if not properly managed. Given the severe impact of these diseases, there has been an increasing use of sensor data to improve the detection and management of these diseases.

RGB sensors have shown considerable potential in detecting fruit and leaf rots, particularly through the use of publicly available datasets such as PlantVillage, which includes images of grapevine diseases such as black rot, esca, and leaf blight, as well as healthy leaves acquired in controlled conditions. Reddy and Neeraja [144] developed an automated system using a DenseNet model, achieving accuracies ranging from 95.57% to 100% for grapevine leaf disease classification. Similarly, Zahra et al. [145] used an Inception-ResNet-v2 for grapevine leaf disease detection, achieving an accuracy of 99.9%. Huang et al. [146] further explored the classification of grapevine diseases and pests (black rot, leaf blight, esca, phylloxera) using an ensemble of CNNs, achieving 100% accuracy, suggesting the potential for real-time intelligent farming systems. Ferentinos [147] evaluated different CNN architectures, with VGG demonstrating the highest performance, reaching a success rate of 99.53% across multiple crop types, including grapevines.

**Figure 10:**
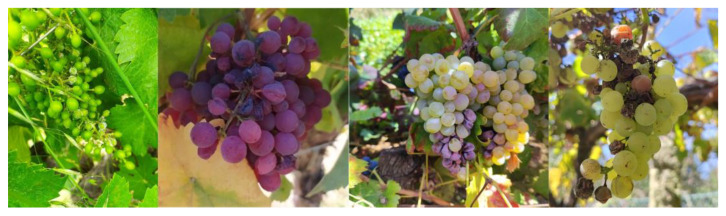
Different types of grape rots, showing different stages of their development.

Proximal imaging platforms have also been adapted for grape bunch analysis. Miranda et al. [148], using the Phenoliner Sensor System equipped with RGB cameras and artificial lighting to detect damaged grapes using a Variational Autoencoder (VAE) with perceptual loss, achieved an accuracy of 92.3% in identifying damaged grapes. Similarly, Strothmann et al. [149] used convolutional autoencoders (CAE) to identify anomalies in grape berries, with the model achieving an ROC-AUC score of 0.95. Both of these studies demonstrate the potential of anomaly detection frameworks for vineyard disease monitoring, such as the examples of the grape rot symptoms presented in Figure 10.

Weather-based models have been developed to predict rot progression. Molitor et al. [82] introduced the BotRisk model, which uses temperature and precipitation data during grape development stages to simulate the thermal-temporal progression of Botrytis bunch rot epidemics with 63% accuracy. The use of these models can bring benefits into analyzing meteorological conditions during critical periods of disease occurrence and progression, with potential applications in other regions, despite limitations from other influencing factors and variations in cultivation practices. In Molitor et al. [26], sigmoidal regression models were used to examine the influence of temperature and precipitation, identifying infection thresholds between 915 and 1222 cumulative degree days (CDD) and linking 5% disease severity to 781.2–1104.7 CDD, demonstrating the role of environmental conditions in bunch rot dynamics.

Other proximity approaches were also evaluated, as in the case of Fuente et al. [78], where the relationship between the normalized difference vegetation index (NDVI) and Botrytis incidence using a GreenSeeker^®^ device (Trimble Agriculture division, Sunnyvale, CA, USA), was analyzed. The authors identified exponential relationships between NDVI values and disease severity, with threshold values between 0.5 and 0.6. The developed regression models (*R*^2^ = 0.74–0.80) highlight the utility of early proximity NDVI monitoring for optimizing fungicide applications. In turn, Rist et al. [150] used point cloud data captured by the Artec^®^ Spider handheld 3D scanner (Artec 3D, Senningerberg, Luxembourg) to predict grape bunch characteristics and disease presence, achieving *R*^2^ values between 0.70 and 0.91 with support vector machines (SVM). This non-invasive method proved suitable for high-throughput phenotyping and disease detection.

UAV-based multispectral data have also shown promise. Ariza-Sentís et al. [151] used UAV imagery to generate NDVI maps and digital elevation models (DEM) for Botrytis bunch rot detection, achieving 70.7% in plant classification using an RF model. Additionally, the study demonstrated a correlation (*R*^2^ = 0.7) between the generated Botrytis bunch rot map values and field data, making it a useful tool to facilitate field inspections and allow different treatments to be prescribed. Vélez et al. [152] used UAV-based MSP data to classify grey rot risk zones, generating products such as DTM, NDVI, CHM, and leaf area index (LAI). Significant differences were found between healthy and botrytis-affected grapevines for all variables (*p*-value < 0.05). An RF model trained on these variables achieved an *R*^2^ of 0.71, providing spatial information for disease management, though the study noted the need for additional variables and seasonal data.

#### 4.3.3. *Flavescence Dorée*

*Flavescence dorée* represents a threat to grapevine cultivation as it may cause significant economic losses. Detection of this disease is challenging, particularly in white grape varieties, where symptoms such as yellowing leaves are less visible compared to the red coloration observed in red grape varieties. Recent advancements in remote sensing and ML techniques have enabled more practical detection methods using data from different sensors.

RGB imaging has been used for detecting *Flavescence dorée* symptoms. Tardif et al. [153] proposed a two-stage detection method using computer vision. RGB images were processed with object detection and segmentation algorithms, including Structure Tensor, ResUnet, and YOLOv4-tiny. Detection accuracy varied by symptom type, with isolated symptom detection accuracy ranging from 0.67 to 0.82 and recall values between 0.39 and 0.59. ResUnet performed well in detecting symptomatic shoots and bunches, achieving precision and recall rates above 0.89 for images acquired under similar conditions as the training data. This method classified images into three categories: *flavescence dorée*, esca, and other diseases. Musci et al. [154] used UAV-based RGB images to classify *flavescence dorée* symptoms. Semantic segmentation using an RF achieved an accuracy of 88%, while Faster R-CNN object detection varied in accuracy from 20% to 82%, depending on configuration. This study highlighted challenges in hyperparameter selection and data scarcity, recommending the use of spectral images for improved detection and identification of disease stages.

HYP and MSP sensors have demonstrated high accuracy in detecting *Flavescence dorée* symptoms through their ability to capture spectral variations. Barjaktarović et al. [39,155,156] developed an MSP and HYP system for detecting symptomatic leaves. These studies used spectral pre-processing techniques such as SNV correction and ML classifiers, achieving classification accuracies above 96%. A low-cost MSP camera proved effective, highlighting its potential as a precision agriculture tool for small farms. Zottele et al. [157] used UAVs equipped with RGB and MSP sensors to compute vegetation indices, such as the chlorophyll index (CI) and normalized difference red edge (NDRE). Near-infrared (NIR) and red-edge bands provided better discrimination between symptomatic and asymptomatic grapevines compared to conventional indices. Similarly, Daglio et al. [158] explored the use of alternative sensors like OptRx^®^ (AgLeader, South Riverside, IA, USA) to calculate NDVI and NDRE, showing that diseased plants exhibited lower values in those indices, although further research is needed to define reliable thresholds.

Feature selection methods have been used to advance *Flavescence dorée* detection. Al-Saddik et al. [159] developed spectral disease indices (SDIs) for *Flavescence dorée* detection, outperforming traditional vegetation indices. In Al-Saddik et al. [160], optimal spectral bands for the design of a dedicated UAV multispectral camera were selected; the authors applied genetic algorithms (GA) and successive projection algorithms (SPA) to select optimal spectral bands. SPA achieved accuracies exceeding 96%, demonstrating their potential for remote disease monitoring and the design of specialized sensors. However, the authors recommended optimizing the technique for each variety and exploring additional methods. In Al-Saddik et al. [161], leaf samples from different varieties were analyzed. Using a spectrometer with a plant probe and an RGB sensor, specific spectral bands (550 nm, 650 nm, and 700 nm) were identified for classification of healthy and infected leaves, achieving an area under the ROC curve (AUC) exceeding 80%. Albetis et al. [162,163] focused on UAV-based MSP data to differentiate symptomatic and asymptomatic grapevines. These studies show that anthocyanin-related indices, such as the Red-Green Index (RGI) and Green-Red Vegetation Index (GRVI), were most effective for red grapevines. For white grapevines, integrating indices in generalized linear models (GLM) improved detection accuracy, with an area under the AUC of up to 0.95. However, challenges in distinguishing *Flavescence dorée* from GTD were reported due to mixed pixels and low infection levels.

Proximal spectroscopy showed effectiveness for early detection of asymptomatic *Flavescence dorée* infections. Imran et al. [164] used portable spectrometers to measure spectral data in the visible and near-infrared ranges. ML models, including logistic regression and SVM, were used. An accuracy of 85% was achieved with ensemble-selected features using an SVM classifier. The feature selection technique proved to be effective in identifying the optimal bands for *Flavescence doreé* detection in vineyards.

Platforms combining sensors and mobile units have facilitated on-the-go disease detection. This is demonstrated in Gallo et al. [165], where the authors developed two mobile prototypes, ByeLab (Bionic eye laboratory) installed on a bins fruit carrier, and an ATV-LAB (utility ATV), for preliminary disease identification in vineyards using LiDAR and MSP sensors. ByeLab, equipped with six MSP sensors, demonstrated higher reliability, correctly identifying low vigor in 80% of cases, compared to 40% by ATV-LAB, which had only two MSP sensors. These systems represent scalable solutions for automated crop monitoring.

#### 4.3.4. Powdery Mildew

Powdery mildew (*Erysiphe necator*) is a fungal disease affecting grapevines, closely resembling downy mildew in its symptoms and target plant organs. Advances in sensor technology, remote sensing, and ML techniques have facilitated the accurate detection and management of this disease.

RGB imaging, combined with deep learning, has demonstrated effectiveness in identifying powdery mildew symptoms. Elsherbiny et al. [166] developed AI GrapeCare, a diagnostic tool using RGB images and hybrid deep networks to detect grapevine diseases, including powdery mildew, black rot, chlorosis, and esca. The system integrated convolutional CNNs, LSTMs, DNNs, and transfer learning architectures (VGG16, VGG19, ResNet50, ResNet101V2). Using textural features extracted via GLCM analysis, the CNN-RGB-LSTM-GLCM model achieved a validation accuracy of 96.6%, with an IoU of 93.4% and a loss of 0.123, demonstrating robust performance in disease diagnosis. Morellos et al. [167] used transfer learning to classify powdery mildew and esca in grapevines. Three CNN architectures—AlexNet, VGG-19, and Inception-v3—were trained on the PlantVillage dataset and a customized dataset. Inception-v3 obtained the highest accuracy, reaching 100% on PlantVillage data and 83.3% on the customized dataset, outperforming AlexNet (87.5% and 66.7%) and VGG-19 (100% and 76.7%).

The integration of IoT systems into vineyard management for powdery mildew monitoring and forecasting was explored. Suciu et al. [168] demonstrated a cloud-based IoT system for machine-to-machine communication in challenging vineyard environments. This system can process Big Data for disease forecasting and generate alerts, showing the potential of IoT applications in improving phytosanitary responsiveness. Oriolani et al. [169] applied logistic regression models based on meteorological data, including thermal-moisture elements recorded by upper canopy sensors, to predict powdery mildew prevalence. Using stepwise variable selection, the models achieved a maximum accuracy of 92.5% in categorizing epidemic severity as severe, moderate, or zero, offering a reliable tool for regional alerts and chemical control strategies. Pero et al. [170] developed regression models using meteorological data and time-domain reflectometry (TDR) probes at varying soil depths. ML algorithms such as RF, extreme gradient boosting (XGBoost), support vector regression, multilayer perceptron, and linear regressions were tested. XGBoost achieved the highest accuracy of 97% for powdery mildew detection, demonstrating the effectiveness of combining IoT data with ML for disease prediction.

Remote sensing techniques, including UAV-based imaging, have also been applied for powdery mildew detection. Li et al. [171] employed UAV RGB imagery to detect and localize grapevine diseases such as powdery mildew, black rot, esca, and leaf blight. The study explored two network types: Multi-fusion U-Net for segmentation and an improved VGG-19 for localization. The Multi-fusion U-Net achieved segmentation accuracies of 87.50%, 80.00% and 78.75% for various diseases. Despite challenges such as low-light interference and motion blur, the study demonstrated the potential of UAV RGB imaging in real-time disease monitoring.

#### 4.3.5. Esca Complex

The Esca complex, a destructive GTD, poses challenges to viticulture. Symptoms include leaf discoloration, necrosis, and overall plant decline, affecting yield and quality. Advancements in remote sensing, imaging, and ML have improved the ability to detect its symptoms, particularly through the integration of RGB, MSP, and HYP data.

RGB imaging mounted on vehicles has been effectively used to identify Esca symptoms. Rançon et al. [172] demonstrated the potential of this approach using image processing and ML techniques. Simple color-based methods, such as histograms, achieved accuracies of approximately 75%, but advanced techniques, including scale-invariant feature transform (SIFT) and CNNs, improved accuracy to 87% and over 90%, respectively. The highest accuracy, 91%, was achieved using deep features extracted from a MobileNet CNN. Kerkech et al. [35] explored UAV-based RGB imagery combined with different color spaces (e.g., RGB, HSV, LAB, YUV) and vegetation indices such as Excess Green (ExG), Excess Red (ExR), Excess Green-Red (ExGR), GRVI, Normalized Difference Index (NDI), and RGI. Using the LeNet-5 CNN architecture, the combination of ExG, ExR, and Excess Green-Red (ExGR) achieved the highest accuracy of 84%. The authors highlight the potential of UAV-based RGB imagery for vineyard disease detection and the importance of optimizing color space and parameter combinations.

MSP and HYP sensors have been used to detect Esca symptoms through spectral analysis. Daglio et al. [173] used UAV-based MSP data to evaluate the NDVI in Esca-affected grapevines. Significant differences in NDVI values were observed between healthy and severely diseased plants, ranging from 0.71–0.79 for healthy plants to 0.64–0.72 for severely diseased plants in Dolcetto, with similar results for Barbera. However, detecting mildly diseased plants remained challenging, as their NDVI values overlapped with healthy plants. Bendel et al. [174] used both ground-based HYP sensors and UAV-based MSP data for Esca detection over three years. Optimal spectral ranges were identified between 700–1000 nm and 1100–1350 nm. ML models trained with field-annotated data achieved up to 95% classification accuracy, with true positive rates reaching 100%. However, aerial detection models showed variable transferability, with accuracies ranging from 58% to 73%, highlighting the need for further optimization. The authors point out that practical application challenges include optimizing aerial detection and further research into detecting other grapevine diseases and nutritional deficiencies, highlighting the importance of early detection for effective control measures.

**Figure 11:**
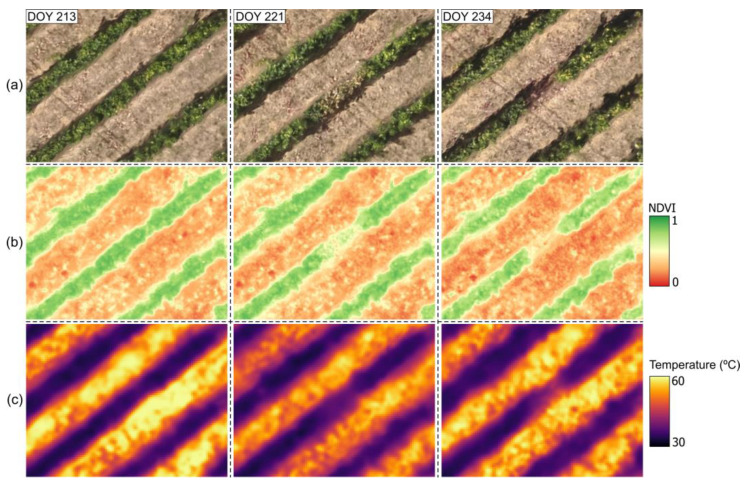
Multi-temporal by Day of Year (DOY) of orthorectified data acquired using unmanned aerial vehicles of a grapevine infected with the esca complex: (**a**) RGB orthophoto mosaic, (**b**) normalized difference vegetation index (NDVI), and (**c**) thermal infrared surface temperature.

The integration of multiple sensors and data sources can be explored for detecting Esca symptoms. Figure 11 illustrates a multi-temporal analysis combining RGB, NDVI, and TIR data, showing the progression of Esca symptoms over time. The use of UAV data in this approach has potential for more comprehensive disease monitoring.

The combination of spectral data and biophysical parameter estimation has proven effective for Esca detection in Al-Saddik et al. [175]. The authors used a portable spectroradiometer and a digital camera with the PROSPECT model inversion to estimate grapevine leaf parameters. ANNs classified between Esca and Yellowing with accuracies ranging from 80.62% to 100% for Esca classification and from 70.45% to 99.54% for yellowing. Combining spectral and textural features improved classification accuracy to over 99% for both diseases, demonstrating the potential of this approach for disease classification.

UAV data combined with climate data have also been applied to monitor Esca disease progression. Di Gennaro et al. [63] used NDVI values and climate indices (Winkler and Huglin) to analyze vegetative state and environmental conditions over two years. Statistical analysis compared remote sensing data with field observations, revealing that the thermal regime was higher in 2012 than in 2013, with more days exceeding 35 °C in the latter year. NDVI values differentiated healthy, symptomatic, and asymptomatic plants, with asymptomatic plants showing intermediate values. The study showed the potential to identify plants at risk of developing disease symptoms based on vegetative state and climate trends.

#### 4.3.6. Viral Diseases

Proximal sensing systems, including handheld spectroradiometers and portable hyperspectral imagers, have demonstrated high accuracy in detecting viral diseases. Wang et al. [176] compared two methods for detecting viral infections in Pinot Noir and Chardonnay grapevines. A proximal spectroradiometer, combined with PLS-DA, achieved an accuracy of 96% for Pinot Noir and 76% for Chardonnay, highlighting the effectiveness of the red-edge spectral region (680–750 nm) for virus detection. Similarly, ELISA laboratory tests confirmed viral infections in 206 of 347 samples, showing the complementary nature of proximal sensing and laboratory diagnostics. Nguyen et al. [31] used hyperspectral imaging to detect early GVCV infections in grapevines. Spectral differences were identified in the 900–940 nm and 400–700 nm ranges, with vegetation indices such as NPQI and FRI1 providing significant discrimination. ML models, including RF and SVM, achieved classification accuracies up to 85.86%, underscoring the potential of hyperspectral imaging for early virus detection. Sinha et al. [177] evaluated VIS-NIR spectroscopy for detecting GLRaV-3 in grapevine leaves across laboratory and field conditions over two seasons. Significant wavelengths (e.g., 1001, 1027, and 1052 nm) were useful for classification, achieving accuracies between 75% and 99%. Quadratic discriminant analysis outperformed Naïve Bayes, confirming the viability of non-destructive spectral methods for early stage virus detection. Similarly, Mehrubeoglu et al. [44] used a portable HYP imaging system to capture leaf images, both in situ and in laboratory conditions. Red blotch disease-affected and healthy areas were manually labeled, and features were extracted, focusing on spectral bands at 566, 628, 680 and 738 nm. An SVM classifier, trained on these features, successfully identified red blotch disease across all images. Disease severity varied, affecting 25% to more than 50% of leaf areas.

RGB imaging, combined with ML techniques, has also been explored for detecting viral diseases in Ampatzidis et al. [178]. The authors used a pre-trained CNN (AlexNet) and a linear SVM to classify images covering six diseases and pests (Black rot, Esca disease, Grapevine yellow, Leaf blight, downy mildew, powdery mildew, and *Stictocephala bisonia*). The system achieved 95.23% accuracy, with a Matthews correlation coefficient of 0.832, demonstrating the feasibility of CNN-based approaches for automating viral disease detection.

**Figure 12:**
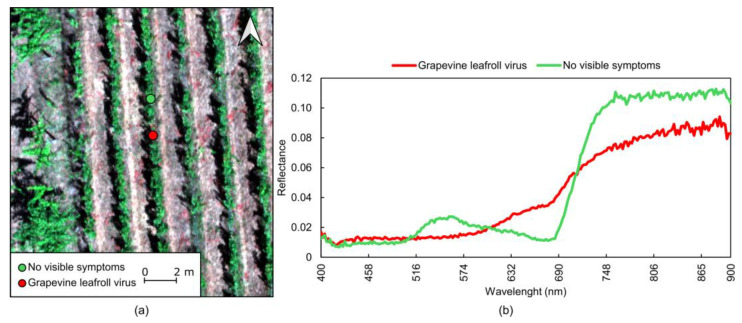
Hyperspectral data acquired from an unmanned aerial vehicle (Headwall Nano-Hyperspec sensor) on a vineyard infected with leafroll virus: (**a**) location of grapevines with and without visible leafroll symptoms; and (**b**) their respective spectral signatures.

Remote sensing techniques, particularly UAV-based hyperspectral imaging, have been used to monitor viral diseases over large vineyard areas. Wang et al. [179] achieved detection accuracies of 98% for red cultivars and 75% for white cultivars using UAV-based hyperspectral imaging and PLS-DA. Spectral indices such as NDVI were also calculated and showed significant correlations with plant health. This approach allows for efficient, rapid monitoring of large vineyard areas, assisting in disease management. An example of a spectral signature from UAV-based HYP data is presented in Figure 12; differences are more evident in wavelengths in the green, red and near infrared regions (Figure 12b).

Wang et al. [180] used UAV-based RGB imagery to monitor Shiraz disease by estimating the projected leaf area (PLA). Virus-infected grapevines showed a lower PLA early in the growing season, with differences of 30–70% compared to healthy plants. A 70% PLA threshold was proposed for classifying infections, providing a cost-effective, rapid monitoring solution.

Airborne hyperspectral imaging systems have been used for virus detection. Rubambiza et al. [32] and Galvan et al. [58] used AVIRIS-NG hyperspectral data and RF classifiers to create risk maps for GLRaV-3 detection [32], achieving accuracies of 84–86%. Savitzky-Golay smoothing and PCA improved classification performance [58]. In turn, MacDonald et al. [181] used airborne HYP imaging with a GIS-based approach to detect GLRaV-3. Detection sensitivity ranged from 88% to over 99%, depending on vineyard-specific factors such as plant age and multiple infections. These studies demonstrate the value of HYP imaging for early detection, though challenges remain in distinguishing between biotic and abiotic stress factors.

## 5. Advances, Limitations and Future Perspectives

Recent advancements in cost-effectiveness and sensor miniaturization, AI, and data processing have contributed to growth in studies on vineyard disease identification and monitoring (Figure 4) [143]. The integration of these tools has enabled researchers to address challenges in viticulture, such as early detection, disease differentiation, and scalability, while maintaining sustainable management practices. Despite this progress, this research field continues to face limitations that require innovative solutions and multidisciplinary approaches.

The detection of diseases in agricultural crops, including vineyards, can be achieved using proximal or remote sensing technologies, mostly when disease symptoms manifest in the canopy (Table 3). Vineyard diseases, whether fungal, bacterial, or viral, can affect leaves, trunks or grapes. Remote sensing using platforms such as UAVs, manned aircraft or satellites and environmental variables from IoT devices and weather stations such as air temperature, humidity, and leaf wetness, enable large-scale disease detection, from identifying conditions favourable for disease occurrence to advanced infection stages. The identification through proximity and remote sensors has become a primary method for early detection [3] and throughout the infection stages [181]. However, implementing IoT devices and their sensors faces challenges such as installation and maintenance costs, technical barriers such as network communication availability and potential damage from agricultural machinery [182]. Additionally, providing a consistent power supply in remote vineyard locations can be difficult, and the reliance on batteries may require frequent replacements or recharging. Environmental factors such as extreme weather conditions can also affect the durability and reliability of IoT devices. Data management and integration with potentially existing vineyard management systems require software and expertise in data analytics, which can be a barrier for smaller vineyards.

TIR sensors are particularly effective in detecting small temperature variations in grapevine leaves caused by fungal infections (Figure 7). However, ground-based TIR data is often limited to specific points and cannot be extrapolated across entire vineyards [125]. On the other hand, UAV-based orthorectified TIR data shows greater potential but may only capture the upper part of the canopy, resulting in less accurate representation of the whole canopy temperature [183] and have a lower spatial resolution compared to other UAV-based sensors. Despite these limitations, TIR data has potential for both early and advanced-stage disease identification (Figure 11c).

Spectral instruments, whether proximal (using spectroradiometers, Figure 9) or remote (using UAV-based HYP data, Figure 12), offer high spectral resolution capabilities across wavelengths from 350 nm to 2500 nm [30]. Optimal results are typically observed between 400 and 1300 nm [174]. These data enable the analysis of spectral changes in fruit and leaves due to disease onset. Although less commonly used for vineyard diseases compared to common optical sensors (Figure 5), spectroscopy sensors have demonstrated potential in other crops [184,185,186] and are being used for the development of spectral indices aimed at disease identification in the field [177].

MSP sensors have shown effectiveness in detecting grapevine diseases, particularly in the blue, NIR, red, and red edge bands [39]. The acquired data can be used to calculate indices such as NDVI, GNDVI, RGI, GRVI, NDRE, and CI [162], to detect changes between infected and healthy areas (Figure 11b). RGB imagery, widely used for vineyard disease detection [128,132,162], is effective in identifying visible symptoms (Figure 8) that can be used to train deep learning models and to calculate indices based on red, green, and blue bands [35]. IoT technologies are being used to detect and predict conditions indicative of fungal disease infections, combining sensor data with ML as a tool to assist winegrowers [143,187]. Fluorescence field data, although in an early applicability stage, shows potential for disease detection in vineyards [131].

The reviewed studies show that current research extensively focuses on detecting fungal diseases, using different methods, including IoT [142,168,169,170], remote, and proximal sensing [42,140] (Figure 6b). IoT WSNs and weather stations have shown potential for early and accurate disease detection, contributing to a reduction in chemical use and improved vineyard management practices. However, challenges such as extreme weather and infrastructure costs must be addressed to ensure long-term sustainability. ML methods have demonstrated high accuracy in quantifying fungal diseases, highlighting the need for additional sensor use [127] and adaptation to local contexts. The combination of computer vision with ML or deep learning techniques has proven effective in detecting and differentiating grapevine diseases, though challenges such as the need for extensive training data and manual pre-processing remain obstacles to fully automating the detection process [129].

Remote sensing approaches, particularly those based on image processing and deep learning, show great potential for rapid diagnoses in short time periods. However, further research is required to develop independent software and quantitatively evaluate these models’ performance [38]. Satellite imagery offers the potential for detecting agricultural diseases but is limited by operational challenges such as cloud cover and, for freely available platforms, spatial, spectral and temporal resolutions [32] (Table 1). The integration of environmental and UAV data offers promising opportunities for detecting diseases, but it is important to consider the accuracy of ground-truth data and the similarities between disease symptoms, nutritional deficiencies in other plants, and canopy density variability (Figure 3). This integration may provide a more complete vineyard overview, but challenges such as image quality and scalability in complex terrains remain [138]. Forecasting models based on meteorological data could complement epidemic observations by providing regional alerts for disease control and prevention. However, further research is needed to improve forecast accuracy given the uncertain correlation between meteorological variables and disease incidence rates [139].

The detection of viral diseases in grapevines has benefited from advances in both proximal [44] and remote sensing [32,188] technologies. Spectroscopy data analysis has demonstrated success in identifying viral infections [176]. MSP sensors, despite challenges in detecting viral infections, have emerged as a diagnosis alternative, but detecting viral infections in white and red grapes remains difficult [133]. UAV-based HYP imaging has shown potential for early detection and monitoring of diseases such as GLRaV-3, with specific wavelengths enabling the identification of symptoms at early stages [181]. Deep learning technologies have been employed to improve disease classification [178,189]. However, the co-occurrence of biotic and abiotic stresses can interfere with the accuracy of the methods. The combination of remote and proximal sensing, along with the integration of optical and other sensors into mobile platforms, enables near real-time monitoring of grapevine health. Studies suggest robust and cost-effective approaches for detecting diseases such as yellows, although continued validation across different conditions and refinement of models are needed [175].

The detection of bacterial grapevine diseases has made progress with proximal and remote sensing technologies. Cost-effective MSP or HYP sensors, integrated with AI approaches, are effective in detecting *Flavescence doreé* at early stages [155]. Image processing techniques and classifiers, such as LDA and CNNs, have demonstrated high accuracy in identifying infected grapevines, despite costs and field condition limitations [159,160]. Optical-based sensors and remote sensing platforms, including UAV-based MSP data [162], enable faster and more complete vineyard data collection. Optimising vegetation indices can improve UAV-based data accuracy in detecting *Flavescence dorée* [163]. Future trends should focus on developing more affordable and efficient sensors and integrating emerging technologies such as LiDAR [165] and AI platforms [166], to reduce costs, improve accuracy, and facilitate the implementation on small-scale farms. Continued research for the adaptation of sensing techniques to different grapevine varieties, training systems, and environmental conditions is essential for ensuring the sustainability and effectiveness of vineyard disease detection in different wine regions.

Cost-effectiveness and scalability of the different sensing approaches are critical factors in the adoption of these technologies for grapevine disease detection. IoT devices, depending on the sensors used, offer continuous monitoring of vineyard environmental conditions at reduced costs, making them suitable for large-scale operations. RGB cameras on smartphones provide a low-cost solution for disease detection through applications developed for the effect. Spectroradiometers, although more expensive, deliver high-spectral resolution data for discriminatory analysis, beneficial for early disease detection. The initial sensor cost can be justified by the potential savings in early disease management and improved yield quality. Optical sensors installed on agricultural machinery enable data collection over large areas, making them suitable for commercial vineyards and to analyze row variability. UAV-based data can offer high-resolution data acquisition capabilities for plant-level analyses (Table 2), which can be scaled to monitor extensive vineyard areas. Although the initial investment is high, the detailed data products can lead to better disease management and reduced chemical use, improving long-term cost-effectiveness. For vineyard-scale analysis, freely available satellite remote sensing data provides a cost-effective option for large-scale monitoring with acceptable revisit periods. This approach is economically viable for large vineyards due to low operational costs and broad coverage.

Therefore, while some approaches, such as UAVs and spectroradiometers, require a higher initial investment, their long-term benefits in improving vineyard management and disease mapping can lead to significant economic gains. On the other hand, freely available satellite data and smartphone-based applications are more affordable options. In terms of operability, the integration of data interpretation services into a single platform would be an ideal solution. This platform could provide vineyard variability maps from earth observation platforms, alerts of ideal conditions for fungal disease occurrence based on IoT devices installed in the field and/or in weather predictions, coupled with algorithms that can interpret different sensor data—whether from spectroradiometers, smartphones, or UAVs—to provide predictive disease diagnostics.

One of the crucial steps in disease detection using sensor data is the creation of datasets to provide the tools for generalized training of AI-based models, with some already available [190,191,192,193]. One challenge is the lack of studies analysing the temporal nature of data from multiple sensors, which could provide perspectives into the spectral, structural or thermal behaviour of diseases throughout the vegetative cycle. Fixed platforms installed within vineyards for continuous monitoring, incorporating various sensors [194], or developing robotic platforms [195,196,197], offer promising short-term alternatives for vineyard disease detection. UAVs at low flight heights, combined with AI methodologies, can facilitate the acquisition of precise data on grapevine disease incidence [198]. Although research in this topic is limited, integrating these sensors with mobile platforms and AI holds potential for advancing disease detection in viticulture.

## 6. Conclusions

This review article presents a comprehensive overview of studies from 2008 to 2024 on using remote and proximal sensing techniques to investigate grapevine diseases. Over the 16-year period, there has been a particular focus on new technologies to monitor diseases in grapevines. Early studies relied on using proximal approaches due to their precision and ease of use. In the last decade, there has been an increase in the use of UAVs that acquire high-resolution data over large areas, using a wide range of sensors across different parts of the electromagnetic spectrum. The integration of proximal and remote sensing represents an advance in disease detection, increasing the potential for automation in vineyard disease identification. This technological progress facilitates faster, more accurate diagnoses, benefiting vineyard management and grapevine health.

Grapevine diseases are identified through a variety of techniques and sensors, with the most common diseases explored being those that manifest visible symptoms on leaves, such as discoloration, necrosis, or other abnormalities. While the identification of leaf symptoms is an important diagnostic tool, relying solely on them can lead to inaccuracies due to the similarity among them, necessitating field confirmation for accurate disease identification. Modern equipment to identify diseases adds value to viticulture in the medium and long term. However, these technologies are still emerging as important tools in widespread disease detection. Consequently, further research is needed to develop platforms that integrate sensor data with AI techniques for improved disease identification, whether using remote sensing platforms, robotic equipment, or agricultural machinery. Additionally, there is a pressing need for the aggregation of data, historical time-series, and data fusion to improve the effectiveness of these technologies and create a robust framework for real-time disease management.

## Figures and Tables

**Table 1:** Remote and proximal sensing platforms used for the detection and monitoring of grapevine diseases.

Platform	Spatial Coverage	Availability	Costs	Capacity
Satellite Platforms	>10 m	Regular revisit periods	Low	Broad overview of vineyard conditions
Manned Aircrafts	Sub-meter	Subject to logistical limitations	High	Detailed capture of smaller areas
Unmanned Aerial Vehicles	Very high (cm level)	On-demand, flexible	Moderate	High-resolution but limited spatial coverage (vineyard plot level)
Weather Stations and IoT Devices	Varies by number of sensors/devices	Continuous, real-time data	Varies	Focused on specific environmental parameters
Proximity Sensing Platforms (Agricultural Machinery, Smartphones)	Plant and leaf level	As needed during operations	Low to Moderate	Continuous, real-time data collection during operations

**Table 2:** Data acquisition sensors for monitoring grapevine diseases and their general viticulture applications.

Sensor Type	Spectrum/Measurement	Applications in Viticulture
RGB	Visible Spectrum	Monitoring leaf color, density and general growth assessment
Thermal Infrared	Thermal Infrared Radiation (8 to 14 µm)	Water stress detection; irrigation scheduling; canopy temperature monitoring
Multispectral	Visible to Near-Infrared (0.4 to 1 µm)	Plant vigor and health assessment; nutrient management; yield estimation; water management
Spectroscopic Equipment	Wide range of wavelengths	Grape chemical composition and ripeness; variety classification; water status; detecting nutrient deficiencies
Weather and Environmental Sensors	Climate, plant, and soil parameters	Real-time vineyard microclimate and environmental monitoring; irrigation support

**Table 3:** Symptoms, modes of transmission or occurrence, primary affected plant parts, and management practices for the studied grapevine diseases. GLRaV: Grapevine Leafroll-associated Virus; GVA: Grapevine Virus A; GVCV: Grapevine Vein Clearing Virus.

Pathogen Type	Disease	Main Symptoms	Spreading/Occurrence Condition(s)	Management Practices	Main Affected Plant Parts
Fungal	Downy Mildew	Oily spots, necrotic lesions, white sporulating layer, shriveling clusters	Humid, mild temperatures, leaf wetness	Fungicides, improve air circulation, monitoring climatic conditions	Leaves, stems, grapes
	Powdery Mildew	White-grayish powdery coating on leaves and berries	Warm, dry climates with high humidity		
	Grey Rot	Brown spots on flowers and leaves, grey sporulation on berries	High humidity, precipitation	Fungicides, removal of infected material	Grapes, flowers, leaves
	Black Rot	Brown spots with black borders on leaves, black spots on berries	Warm, humid climates	Removal of infected plant debris, fungicides, pruning	Leaves, grapes
	Sour Rot	Vinegary odor, berry splits	High humidity during ripening	Removal of infected material, insect vector control	Grapes
	White Rot	Watery rot in grapes	High humidity	Removal of infected material, fungicides	
	Esca Complex	Chlorotic streaks, vascular discoloration, necrosis, apoplexy	Pruning activities or plant damage	Prevent pruning wounds, remove infected plants	Trunk, leaves
Bacterial	*Flavescence Dorée*	Yellowing and curling leaves, stunted shoots, necrosis of inflorescences	Leafhopper	Insect vector control, remove infected plants	Leaves, shoots, inflorescences
Viral	GLRaV	Leaf discoloration, rolling, stunted growth, reduced yield	Climatic conditions, insect vectors, grafting	Use of virus-free planting material and grafting practices, control insect vectors, management of rugose wood complex	Leaves, grapes, trunk, vascular tissues
	GVA	Bark cracking, vascular tissue damage, reduced yield			
	GVCV	Vein clearing in leaves, deformities, decreased vigor			

## Data Availability

The original contributions presented in this study are included in the article/Supplementary Materials. Further inquiries can be directed to the corresponding author.

## Supplementary Materials

The following supporting information can be downloaded at: https://www.mdpi.com/article/10.3390/s24248172/s1, Table S1. PRISMA 2020 for abstracts checklist. Table S2. PRISMA 2020 checklist. Table S3. Databases and search queries used in the systematic review.
